# Enterovirus Infection Restricts Long Interspersed Element 1 Retrotransposition

**DOI:** 10.3389/fmicb.2021.706241

**Published:** 2021-10-18

**Authors:** Yan Li, Siyu Shen, Haoran Guo, Zhe Zhang, Lili Zhang, Qingran Yang, Yanhang Gao, Junqi Niu, Wei Wei

**Affiliations:** ^1^Institute of Virology and AIDS Research, First Hospital, Jilin University, Changchun, China; ^2^Key Laboratory of Organ Regeneration and Transplantation of Ministry of Education, Institute of Translational Medicine, First Hospital, Jilin University, Changchun, China; ^3^Department of Hepatology, First Hospital, Jilin University, Changchun, China

**Keywords:** enterovirus, LINE-1, EV-D68, EV-A71, retrotransposon

## Abstract

Long interspersed element 1 (LINE-1 or L1) is the only active autonomous retrotransposon in the human genome that can serve as an endogenous upstream activator of cytoplasmic nucleic acid sensing pathways to elicit an antiviral immune response. In this study, we investigated the influence of enteroviral infection on L1 mobility. The results showed that infection with different enteroviruses, both EV-D68 and EV-A71, blocked L1 transposition. We screened diverse viral accessory proteins for L1 activity and identified EV-D68 2A, 3A, 3C, and EV-A71 ORF2p proteins as viral L1 inhibitors. EV-D68 2A suppressed L1 mobility by expression suppression of L1 proteins. Viral proteins 3A and 3C restricted ORF2p-mediated L1 reverse transcription in isolated L1 ribonucleoproteins. The newly identified enteroviral protein ORF2p inhibited the expression of L1 ORF1p. Altogether, our findings shed light on the strict modulation of L1 retrotransposons during enterovirus replication.

## Introduction

Transposable elements have been recognized as major contributors to mammalian genomes since the discovery of mobile DNA (O’Donnell and Burns, 2010). They are categorized into retrotransposons and DNA transposons. Retrotransposons include non-long terminal repeat (non-LTR) elements and long terminal repeat (LTR). Human long interspersed nuclear element 1 (LINE-1 or L1), the only autonomously retrotransposition-competent retrotransposon (Scott et al., 1987; Kazazian et al., 1988), constitutes the most abundant family of autonomous retroelements in mammals. Human L1 encodes two proteins, ORF1p and ORF2p. L1 ORF1p is a RNA-binding protein of about 40 kDa with nucleic acid chaperon activity, and L1 ORF2p is a protein of about 150 kDa with both endonuclease and reverse transcriptase (RT) activity (Belancio et al., 2008; Kopera et al., 2016). L1 proteins interact with L1 RNA to facilitate the assembly of L1 ribonucleoproteins (RNPs). The entry process by which L1 RNPs move from the cytoplasm into the nucleus is dependent on the nuclear localization signal on L1 ORF1p (Freeman et al., 2019). Upon entering the nucleus, L1 ORF2p uses its nuclease activity to form a single chain gap on genome DNA, and then uses its RT activity, with the single chain gap DNA as the primer and L1 mRNA as the template, to start the reverse transcription (Kazazian and Moran, 1998; Goodier, 2016). Target-site-primed reverse transcription (TPRT) generates a new copy of L1s (Kazazian and Moran, 1998; Goodier, 2016). This process is followed by integration events, such as non-homologous end-joining repair (Suzuki et al., 2009).

L1 retrotransposition is largely restricted in normal tissues but can be activated in response to stress, aging, or disease (Beck et al., 2011; Goodier, 2016; Liu et al., 2018). Recently, HCV was found to restrict human L1 retrotransposition in hepatoma cells (Schobel et al., 2021). Previous studies have also uncovered upregulated endogenous L1 and other transposon levels by virus infection (Geuking et al., 2009; Horie and Tomonaga, 2011; Iijima et al., 2013; Jones et al., 2013; Nakayama et al., 2019; Yin et al., 2021; Zhang et al., 2021). L1 is a major source of insertional mutagenesis in cells. L1 can also serve as an endogenous activator of the antiviral immune response through the cGAS-STING or RIG-I/MDA5-MAVS sensing pathways (Zhao et al., 2018). Abnormal L1 activity induces type-I interferon (IFN-I) production in aged cells and is associated with the progression of inflammatory diseases (Liu et al., 2018; De Cecco et al., 2019; Simon et al., 2019).

Enteroviruses (EVs) of the Picornaviridae family, small, non-enveloped, icosahedral viruses which comprises non-enveloped RNA viruses, provoke a diverse array of clinical symptoms (Baggen et al., 2018). The genome of EVs is a positive-sense single-stranded RNA genome, including two open reading frames (ORFs), 5’ untranslated region (UTR), and 3’ UTR. The major ORF encodes a polyprotein that hydrolyzes into four structural proteins (VP1, VP2, VP3, and VP4) and seven non-structural proteins (2A, 2 B, 2C, 3A, 3B, 3C, and 3D). Recently, we and others have identified another ORF harbored upstream (ORF2/uORF) (Guo et al., 2019; Lulla et al., 2019). The translation product ORF2p/UP is required for viral replication in intestinal epithelial cells. Accumulating evidence indicates that diverse interferon-stimulated proteins maintain potent antiviral activity against enteroviruses. A series of enteroviral proteins target diverse components of nucleic acid-sensing pathways to suppress host immune activation (Xiang et al., 2014, 2016; Rui et al., 2017). However, the influence of enterovirus infection on the host innate immune system’s endogenous activator, LINE-1, is unclear. Hence, in the present study, we investigated the cross-talk between enterovirus and L1.

## Materials and Methods

### Cells and Plasmids

HEK293T cells were cultured in Dulbecco’s modified Eagle’s medium (DMEM) supplemented with 10% fetal bovine serum and 100 μg/mL penicillin-streptomycin. Human LINE-1 constructs 99 PUR L1RP EGFP, 99 PUR JM111 EGFP (JM111), the pc-L1-1FH plasmid, and pEGFP-N1-ORF1-EGFP plasmids were kindly provided by Dr. Haig H. Kazazian, Jr., and John L. Goodier (Moran et al., 1996; Ostertag et al., 2000; Goodier et al., 2012). The JM111 construct containing two missense mutations in the ORF1 region was used as a negative control for L1 EGFP. The pc-L1-1FH plasmid expressed full-length LINE-1, and LINE-1 ORF1p was tagged with FLAG and HA (Goodier et al., 2012). The pYX014 and pYX017 plasmids were kindly provided by Dr. Wenfeng An (Xie et al., 2011; Kawano et al., 2018). EV-D68 2A, 2B, 2C, 3A, 3C, 3D, and EV-A71 ORF2p expressing vectors were cloned into VR1012 vector and tagged with HA-. The empty vector VR1012 was generously provided by Vical (San Diego, CA, United States), and the control vector pcDNA 3.1(+) plasmid was purchased from Invitrogen Corporation (Carlsbad, CA, United States).

### Viruses

EV-D68 prototype Fermon (2014) isolated US/MO/14-18947 (MO) and US/KY/14-18953 (KY) were purchased from ATCC. Professor Cheng Tong kindly provided the EV-A71 prototype Anhui2007, and prototype CC063 was isolated in our laboratory. Viruses propagated in RD cells. The supernatants of enterovirus-infected cells were harvested, filtered through a 0.22 mm filter, and centrifuged through a 20% sucrose cushion in an SW28 rotor at 28,000 rpm for 1.5 h. Purified pellets were stored at −80°C.

### Long Interspersed Element 1 Retrotransposition With EGFP Reporter Assay

HEK293T cells were seeded in 24 well plates and transfected with 1 μg of L1RP EGFP or JM111 constructs. Cells were then puromycin-selected (3 μg/mL) 48 h post-transfection. The percentage of GFP(+) cells was measured using a BD FACSCalibur Flow Cytometer after 48 h of puromycin selection. The plasmid JM111 construct was used as a negative control. A total of 10,000 single-cell events were gated using flow cytometry. The percentage of GFP(+) cells was analyzed using the CellQuest Pro (v.5.2).

### Long Interspersed Element 1 Retrotransposition With a Luciferase Reporter System

HEK293T cells seeded in 24-well plates were transfected with the pYX014 or pYX017 plasmids. The cells were then puromycin selected 24 h post-transfection. After 3 days of puromycin selection, dual-luciferase assays were performed according to the manufacturer’s instructions (Promega). Firefly and Renilla luciferase were measured using Promega GloMax^®^ (Sunnyvale, CA, United States) from a single sample.

### Cell Viability Assay

HEK 293T cells were pre-infected or pre-transfected. The cells were seeded in 96-well plates 1 day post infection/transfection. Then the cells were cultured for 3 days before assessing thier viability. Absorbance was detected at a wavelength of 490 nm using a BioTek ELISA reader (BioTek Instruments, Inc.) on adding 3-(4,5-dimethylthiazol-2-yl)-5-(3-carboxymethoxyphenyl)-2-(4-sulphophenyl)-2H-tetrazolium, inner salt (MTS) (Promega, United States) to each well.

### Immunoblotting

Cell samples were harvested and lysed in RIPA buffer (1 M Tris pH 7.8, 1M NaCl, 1% NP-40, 0.5 M EDTA). The cell lysate was separated on 12% SDS-PAGE gels and then transferred to nitrocellulose membranes using a semidry apparatus (Bio-Rad). Anti-HA was purchased from Thermo Fisher Scientific (Waltham, MA, United States). The anti-GAPDH antibody and anti-α-Tubulin antibody were purchased from GenScript Biotech Corp (NJ, United States). Anti-EV-D68 VP1 antibody and anti-EV-A71 VP1 antibody were purchased from GeneTex (San Antonio, TX, United States).

### Immunostaining and Fluorescence Imaging

HEK 293T cells were transfected with pEGFP-N1-ORF1-EGFP plasmids in the absence or presence of enterovirus protein-expressing plasmids (HA-tagged). After 2 days, cells were fixed with 4% paraformaldehyde in PBS for 30 min, permeabilized in 0.1% Triton X-100 in PBS for 10 min, and blocked in 5% BSA solution for 1 h. Then, the cells were incubated with anti-HA antibody overnight at 4°C. Cells were incubated with Alexa Fluro 594 conjugated goat anti-rabbit IgG antibody (Life Technologies, A-11012) for 1 h at 4°C. Nuclei were counterstained with 4’6-diamidino-2-phenylindole (DAPI). Fluorescence imaging was performed using a fluorescence microscope (OLYMPUS) with a maximum magnification of 40 X.

### LEAP Assay and Quantitative Real-Time PCR

The L1 construct pc-L1-1FH has been described (Goodier et al., 2012). HEK293T cells were transfected with the pc-L1-1FH plasmid. L1 RNPs were separated by sucrose cushion of 8.5 + 17% gradient centrifugation at 4°C, 178,000 g for 2 h as previously described (Liang et al., 2016). During the LEAP assay, 2 μL of the L1 RNP sample was added to each cDNA extension reaction solution [500 mM KCl, 50 mM MgCl_2_, 500 mM Tris–HCl (pH 7.5), 1M DTT, RNasin (40U/μL), 0.05%(v/v) Tween 20, and dNTP] using the LEAP primer: 5′-GCGAGCACAGAATTAATACGACTCACTATAGGTTTTTTT TTTTTVN-3′. L1 mRNA was reversed-transcribed to L1 cDNA by L1 ORF2p in the RNPs at 37°C for 1 h. To detect the level of the L1 mRNA, L1 RNA was extracted from the L1 RNP and reverse-transcribed with the same primer using MuLV RT (GoScript Reverse Transcription System, Promega). The synthesized cDNA from both LEAP assay and MuLV RT was then analyzed by quantitative real-time PCR (qRT-PCR) using the following primers: linker PCR primer, 5′-GCGAGCACAGAATTAATACGACT-3′; L1 3′-end primer, 5′-GGGTTCGAAATCGATAAGCTTGGATCCAGAC-3′, with a standard three-step method (95°C for 15 s, 60°C for 1 min, and 72°C 2–4 kb/min) as previously described (Liang et al., 2016). The 2^–Δ^ ^Δ^ ^CT^ method was used for calculations.

### RNA Quantitation by Quantitative Real-Time PCR

Total RNA from the samples of interest was first isolated using TRIzol (Life Technologies) and then subjected to reverse transcription using MonScript RTIII All-in-One Mix with dsDNase according to the manufacturer’s instructions. The qRT-PCR was performed using MonAmp^TM^ ChemoHS qPCR Mix and specific primers. The reaction was performed under the following conditions as suggested by the manufacturer: 95°C for 10 min; 40 cycles of 95°C for 10 s, 60°C for 10 s and 72°C for 1 min; and a dissociation protocol. The primers used for qRT-PCR were as follows: L1 ORF1p, forward (5′-CAAACACCGCATATTCTCACTCA-3′) and reverse (5′-CTTCCTGTGTCCATGTGATCTCA-3′); L1 ORF2p, forward (5′-AGGAAATACAGAGAACGCCACAA-3′) and reverse (5′-GCTGATATGAAATTCTGGGTTGA-3′); GAPDH, forward (5′-GCAAATTCCATGGCACCGT-3′) and reverse (5′-TCGCCCCACTTGATTTTGG-3′); EV-D68, forward (5′-TGTTCCCACGGTTGAAAACAA-3′) and reverse (5′-TGTCTAGCGTCTCATGGTTTTCAC-3′). Endogenous mRNA levels of GAPDH were used as the loading control and are not shown unless otherwise indicated.

### Statistical Analysis

This study’s statistical data were analyzed using GraphPad Prism software (version 8.0; GraphPad Software Inc.). Data are described as the M ± SD from three replicates of each experiment. Unpaired Student’s *t*-test was performed on the data between the two groups. *P* < 0.01 was considered significant in groups.

## Results

### Enterovirus Suppresses Human Long Interspersed Element 1 Retrotransposition

**FIGURE 1:**
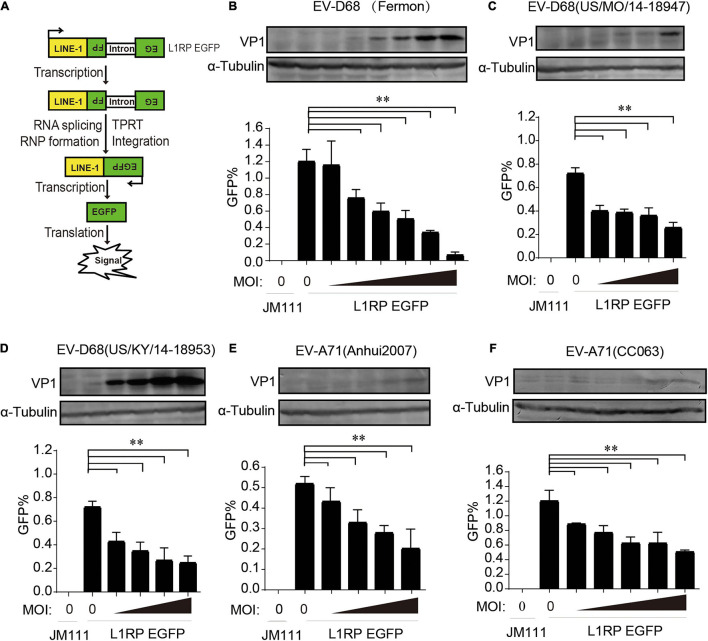
Suppression of long interspersed element 1 (LINE-1) retrotransposition by both EV-D68 and EV-A71. **(A)** Retrotransposition assay process. EGFP can only be detected when LINE-1 successfully transposes into the genome and the intron is removed during RNA splicing. **(B–D)** L1RP EGFP plasmids were transfected into HEK293T cells. Cells were then infected with EV-D68 (Fermon) at increasing MOI of 0, 2 × 10^–5^, 5 × 10^–5^, 1 × 10^–4^, 2 × 10^–4^, 5 × 10^–4^, and 1 × 10^–3^ or EV-D68(US/MO/14-18947)/EV-D68(US/KY/14-18953) at increasing MOI of 0, 2 × 10^–5^, 5 × 10^–5^, 1 × 10^–4^, and 2 × 10^–4^ 12 h post-transfection. Retrotransposition-defective LINE-1 (JM111) was used as a negative control. 4 days after transfection, flow cytometry assay was performed to determine EGFP-positive cells. The bar graph of EGFP-positive cells represents retrotransposition efficiency. The asterisks indicate statistically significant differences between groups by Unpaired Student’s *t*-test (*p* < 0.01). After flowcytometry assay, the cells were harvested, lysed and immunoblotted by anti-EV-D68 VP1 antibody and anti-α-Tubulin antibody. **(E,F)** HEK293T cells were transfected with L1RP EGFP plasmids and then infected with EV-A71(Anhui2007) at increasing MOI of 0, 2 × 10^–4^, 5 × 10^–4^, 1 × 10^–3^, and 2 × 10^–3^ or EV-A71(CC063) at increasing MOI of 0, 1 × 10^–3^, 2 × 10^–3^, 5 × 10^–3^, 1 × 10^–2^, and 2 × 10^–2^ 12 h post-transfection. JM111 was used as a negative control. Flow cytometry assay was performed to determine EGFP-positive cells. The bar graph of EGFP-positive cell fraction represents retrotransposition efficiency. The asterisks indicate statistically significant differences between groups by Unpaired Student’s *t*-test (*p* < 0.01). After flowcytometry assay, the cells were harvested, lysed and immunoblotted by anti-EV-A71 VP1 antibody and anti-α-Tubulin antibody. All experiments in this figure were performed in triplicate, and each error bar indicates the SD of three replicates in one experiment.

We used a well-established EGFP reporter system in HEK293T cells to evaluate the effect of enterovirus infection on L1 retrotransposition (Moran et al., 1996; Ostertag et al., 2000; Goodier et al., 2012). The L1RP EGFP plasmids contained L1RP and an EGFP reporter cassette interrupted by introns. The EGFP cassette had its CMV promoter. EGFP signals can only be detected after successful intron removal and L1 integration (Figure 1A). The retrotransposition-defective JM111 plasmids contained two missense mutations in the ORF1 region and were used as negative controls (Moran et al., 1996; Ostertag et al., 2000; Goodier et al., 2012). The pL1RP-EGFP-transfected HEK293T cells were infected with EV-D68 [prototype Fermon, 2014 isolated US/MO/14-18947 (MO) and US/KY/14-18953 (KY), respectively]. GFP-positive cells’ ratio was analyzed by flow cytometry, and this indicated that all EV-D68 strains potently suppressed L1 retrotransposition compared with that by the control group in a dose-dependent manner (Figures 1B–D and Supplementary Figures 1A–C). Furthermore, we investigated the role of EV-A71 infection in L1 mobility and showed that two EV-A71 (Anhui2007, CC063) strains strongly suppressed the EGFP-based L1 retrotransposition activity compared with that by control (Figures 1E–F and Supplementary Figures 1D,E). We then measured the cytotoxicity of all the enterovirus strains in HEK293T cells to exclude any possible toxic effects of enterovirus that would bias the results. None of the indicated multiplicity of infection (MOI) of enteroviruses was toxic to HEK293T cells (Supplementary Figures 2A,C,E,3A,C). Moreover, these enteroviruses did not interrupt the expression of EGFP driven by the CMV promoter in pcDNA3.1-EGFP-transfected HEK293T cells (Supplementary Figures 2B,D,F,3B,D). In RD and A549 cell, endogenous L1 mRNA levels were efficiently inhibited by EV-D68 infection (Supplementary Figure 4). Further, EV-D68 also inhibited L1-triggered RLR-mediated IFN-β production (Supplementary Figure 5). Hence, enteroviral infection limits intracellular L1 mobility.

### Enterovirus Non-structural Proteins Abrogate Long Interspersed Element 1 Retrotransposition Activity

**FIGURE 2:**
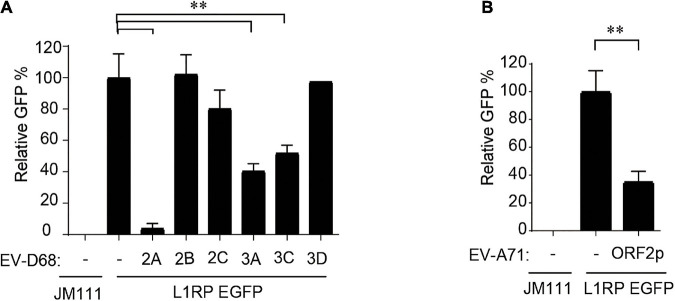
Non-structural proteins of enterovirus impact on LINE-1 retrotransposition. **(A,B)** Empty vector VR1012 or EV-D68 2A, 2B, 2C, 3A, 3C, 3D, EV-ORF2p-expressing plasmids were co-transfected into HEK293T cells with L1RP EGFP plasmids. JM111 was used as a negative control for the cytometry assay. The bar represents retrotransposition efficiency, with the VR1012-transfected sample set to 100%. The asterisks indicate statistically significant differences between groups by Unpaired Student’s *t*-test (*p* < 0.01). All experiments in this figure were performed in triplicate, and each error bar indicates the SD of three replicates in one experiment.

Subsequently, we used individual EV-D68 non-structural protein-expressing constructs to determine which of the following EV-D68 protein suppresses L1 activity: 2A, 2B, 2C, 3A, 3C, or 3D. These EV-D68 protein-expressing plasmids were co-transfected with pL1-EGFP into HEK293T cells, and GFP-positive cells were determined 96 h after transfection. The results showed that three of these EV-D68 proteins, 2A, 3A, and 3C, significantly reduced L1 mobility (Figure 2A). We identified that the newly identified EV-A71 protein, ORF2p, was another viral inhibitor of L1 transposition activity (Figure 2B). Overexpression of tested viral proteins in HEK293T cells had no apparent toxic effects on cellular viability or did not impact the expression of EGFP driven by the CMV promoter (Supplementary Figures 3E,F).

### Enterovirus 2A Protein Modulates Long Interspersed Element 1 Activity via Long Interspersed Element 1 Expression Inhibition

**FIGURE 3:**
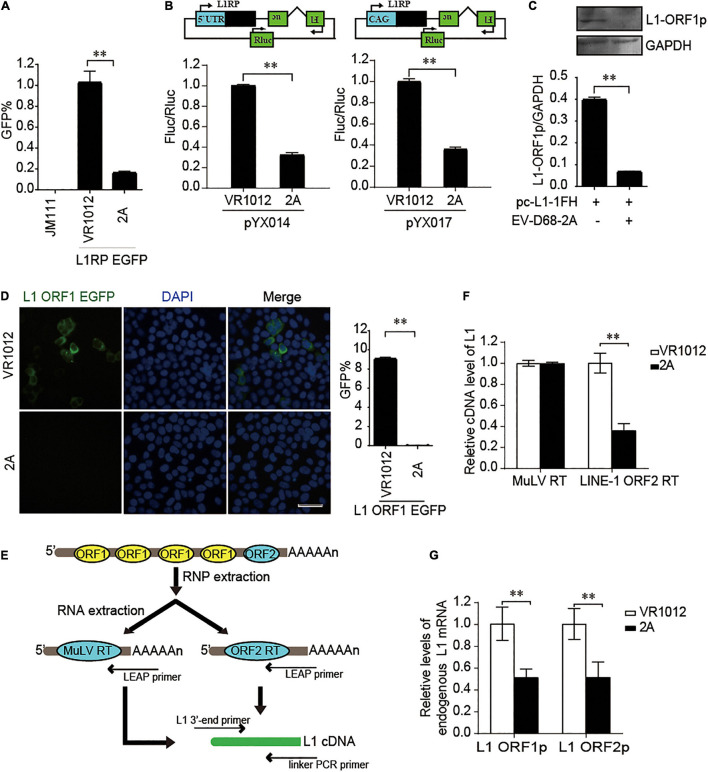
EV-D68 2A modulates LINE-1 retrotransposition in an ORF1p and ORF2p dependent manner. **(A)** L1RP EGFP plasmids and empty vector VR1012 or EV-D68 2A expressing construct were co-transfected into HEK293T cells. JM111 was used as a negative control. Flow cytometry was performed 4th day post-transfection. The bar graph depicts the M ± SD of each experiment, performed in triplicate The asterisks indicate statistically significant differences between groups by Unpaired Student’s *t*-test (*p* < 0.01). **(B)** Empty vector VR1012 or EV-D68 2A expressing construct was co-transfected with the pYX014/pYX017 construct into HEK293T cells. Dual-Luciferase assays were performed according to the manufacturer’s instructions 96 h post-transfection. The bar graph of Firefly and Renilla luciferase ratio represents the retrotransposition frequency, with the VR1012-transfected sample set to 1.0. The asterisks indicate statistically significant differences between groups by Unpaired Student’s *t*-test (*P* < 0.01). **(C)** VR1012 or VR1012-2A plasmids were co-transfected with pc-L1-1FH. Anti-HA antibodies immunoblotted the cell lysate. **(D)** HEK293T cells were transfected with pEGFP-N1-ORF1-EGFP and VR1012 or VR1012-2A plasmids. Immunostaining and fluorescence imaging were performed as described in the “Materials and Methods” 48 h post-transfection: scale bar, 20 μm. Bar graph represents the percentage of GFP-positive cells from flowcytometry. The asterisks indicate statistically significant differences between groups by Unpaired Student’s *t*-test (*p* < 0.01). **(E)** Schematic for LEAP assay. HEK293T cells were transfected with pc-L1-1FH. LINE-1 ribonucleoproteins complexes were created from the nucleofected cells and purified by sucrose cushion centrifugation. LINE-1 cDNA was reverse-transcribed with the assistance of either ORF2p as previously described or MuLV following the manufacturer’s instructions. Synthesized cDNA was then analyzed by quantitative real-time PCR. **(F)** LEAP assay was performed as described in “Materials and Methods.” MuLV RT products and LEAP products were analyzed by Quantitative real-time PCR. The bar graph of relative cDNA level of L1 represents reverse transcription efficiency of L1 ORF2p or MuLV. The relative cDNA level of MuLV RT was set to 1.0. The asterisks indicate statistically significant differences between groups by Unpaired Student’s *t*-test (*p* < 0.01). **(G)** HEK293T cells were transfected with VR1012 or VR1012-2A plasmids. Total RNA was isolated and subjected to reverse transcription. Then, qRT PCR was performed using L1 ORF1p, L1 ORF2p, and GAPDH specific primers. The results were normalized by endogenous levels of GAPDH mRNA. Data are represented as the mean ± SD, with the VR1012 group set to 1.0. The asterisks indicate statistically significant differences between groups by Unpaired Student’s *t*-test (*p* < 0.01).

We further used the dual-luciferase reporter plasmids (pYX014 and pYX017) for L1 retrotransposition (Xie et al., 2011; Kawano et al., 2018) to confirm the influence of EV 2A on L1 activity. The pYX014 or pYX017 plasmids contains both Firefly and Renilla luciferase, which were used as an indication of L1 retrotransposition and for normalization, respectively. The pYX014 construct contains full length L1RP, while the pYX017 construct was generated by replacing the L1 5’-UTR with a strong CAG promoter to increase retrotransposition (Xie et al., 2011; Kawano et al., 2018). Like the EGFP reporter (Figure 3A), relative firefly luciferase activity was decreased by EV-D68 2A (Figure 3B). Next, we examined the effects of 2A on the expression and function of L1 encoded proteins. Immunoblotting (Figure 3C) and immunofluorescence assays (Figure 3D) showed that compared with that by control, 2A more efficiently blocked the expression of L1 ORF1p. Per the effects of 2A on L1 protein expression, we conducted an *in vitro* LEAP reverse transcriptase (RT) assay to assess the function of ORF2p in LINE-1 ribonucleoprotein (RNP) complexes (Figure 3E; Kulpa and Moran, 2006). We found that the L1 RNP RT activity was significantly suppressed in the presence of EV-D68 2A (Figure 3F), which is possibility mainly due to inhibition on the mRNA levels of both L1 proteins (Figure 3G). Collectively, EV-D68 2A directly targets the expression of L1 proteins to suppress L1 mobility.

### EV-D68 3A Protein Restricts the Reverse Transcription Activity of Long Interspersed Element 1 ORF2p

**FIGURE 4:**
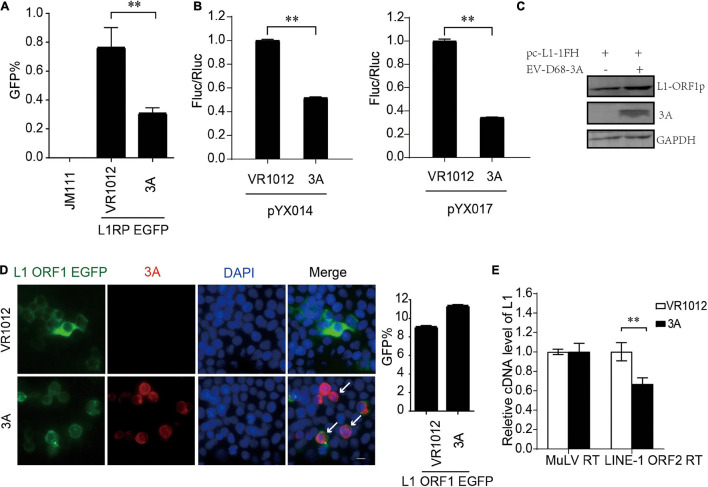
Inhibition of LINE-1 retrotransposition by EV-D68 3A. **(A)** L1RP EGFP plasmids and VR1012/VR1012-3A plasmids were co-transfected into HEK 293T cells. JM111 was used as a negative control. Flow cytometry assay was performed 4 days post-transfection. The bar graph depicts the M ± SD of each experiment, performed in triplicate. The asterisks indicate statistically significant differences between groups by Unpaired Student’s *t*-test (*p* < 0.01). **(B)** pYX014/pYX017 constructs and VR1012/VR1012-3A- plasmids were co-transfected into HEK 293T cells. Dual-Luciferase assay was performed 4 days after transfection. The VR1012-transfected group was set to 1.0. The asterisks indicate statistically significant differences between groups by Unpaired Student’s *t*-test (*p* < 0.01). **(C)** VR1012 or VR1012-3A plasmids were co-transfected with pc-L1-1FH. Immunoblot was performed on cell lysate after 48 h. **(D)** HEK293T cells were transfected with pEGFP-N1-ORF1-EGFP and VR1012/VR1012-3A plasmids. Immunostaining and fluorescence imaging were performed as described in the “Materials and Methods” after 48 h: scale bar, 20 μm. Bar graph represents the percentage of GFP-positive cells from flowcytometry. The asterisks indicate statistically significant differences between groups by Unpaired Student’s *t*-test (*p* < 0.01). **(E)** LEAP assay and quantitative real-time PCR were performed as described in the “Materials and Methods.” The bar graph of relative cDNA level of L1 represents reverse transcription efficiency of L1 ORF2p or MuLV. The relative cDNA level of MuLV RT was set to 1.0. The asterisks indicate statistically significant differences between groups by Unpaired Student’s *t*-test (*p* < 0.01).

We confirmed L1 inhibition by 3A in L1-EGFP and L1-Luciferase assays (Figures 4A,B). We then measured the influence on the expression of L1 ORF1p proteins. Repeated immunoblotting data indicated that 3A, unlike 2A, did not decrease the levels of intracellular ORF1p (Figure 4C). Meanwhile, the immunofluorescence assays indicated that 3A treatment resulted in both L1 ORF1p and 3A localization in the cytoplasm, and there was no observable change in the cytoplasmic localization of L1 ORF1p (Figure 4D). The coimmunoprecipitation results showed that these proteins did not interact (Supplementary Figure 6). However, the presence of 3A significantly suppressed the RT activity of L1 ORF2p in L1 RNPs (Figure 4E). Thus, enteroviral 3A restricts L1 mobility by targeting the reverse transcription step.

### EV-D68 3C Protein Restricts the Reverse Transcription Activity of Long Interspersed Element 1 ORF2p

**FIGURE 5:**
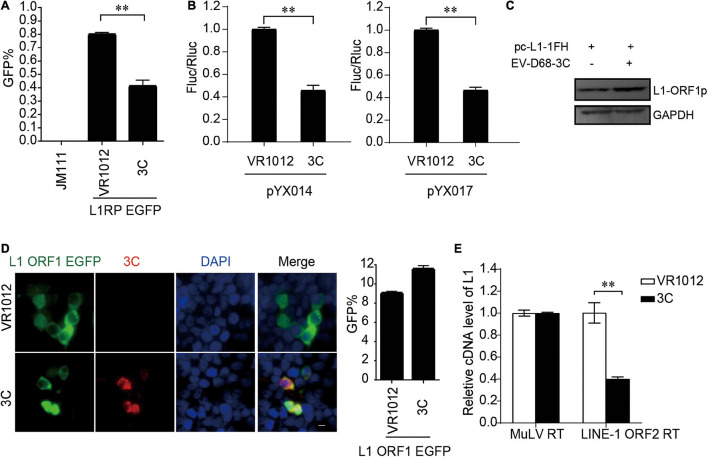
Suppression of LINE-1 retrotransposition by EV-D68 3C. **(A)** L1RP EGFP plasmids and VR1012/VR1012-3C plasmids were transfected into HEK 293T cells. JM111 was used as a negative control. Flow cytometry was performed 96 h post-transfection. The bar graph depicts the M ± SD of each experiment, performed in triplicate The asterisks indicate statistically significant differences between groups by Unpaired Student’s *t*-test (*p* < 0.01). **(B)** HEK 293T cells were transfected with pYX014 or pYX017 constructs together with VR1012 or VR1012-3C-. Then dual-luciferase assay was performed 4 days post-transfection. The VR1012 transfected sample was set to 1.0. The asterisks indicate statistically significant differences between groups by Unpaired Student’s *t*-test (*p* < 0.01). **(C)** VR1012 or VR1012-3C plasmids were co-transfected with pc-L1-1FH. Western blot was performed after 48 h. **(D)** pEGFP-N1-ORF1-EGFP and VR1012/VR1012-3C plasmids were co-transfected. Immunostaining and fluorescence imaging were performed as described in the “Materials and Methods” 48 h post-transfection: scale bar, 20 μm. Bar graph represents the percentage of GFP-positive cells from flowcytometry. The asterisks indicate statistically significant differences between groups by Unpaired Student’s *t*-test (*p* < 0.01). **(E)** LEAP assay and quantitative real-time PCR were performed as described in the “Materials and Methods.” The bar graph of relative cDNA level of L1 represents reverse transcription efficiency of L1 ORF2p or MuLV. The relative cDNA level of MuLV RT was set to 1.0. The asterisks indicate statistically significant differences between groups by Unpaired Student’s *t*-test (*p* < 0.01).

Next, all the L1-EGFP and L1-Luciferase assays demonstrated that 3C robustly impaired LINE-1 mobility (Figures 5A,B). The results from repeated experiments indicated that EV-D68 3C was not detectable by immunoblotting assays. The overexpression of 3C protein did not reduce L1 ORF1p expression (Figure 5C) and showed no influence on the sublocalization of ORF1p (Figure 5D). However, 3C specifically inhibited L1 ORF2p activity in the LEAP assay (Figure 5E), demonstrating that L1 inhibition by 3C targets the reverse transcription process during L1 transposition. In addition, we further confirmed that EV-A71 3C, which can be detected by immunoblotting, maintained a conserved function for L1 transposition (Supplementary Figure 7).

### EV-A71 ORF2p Suppresses Long Interspersed Element 1 Retrotransposition

**FIGURE 6:**
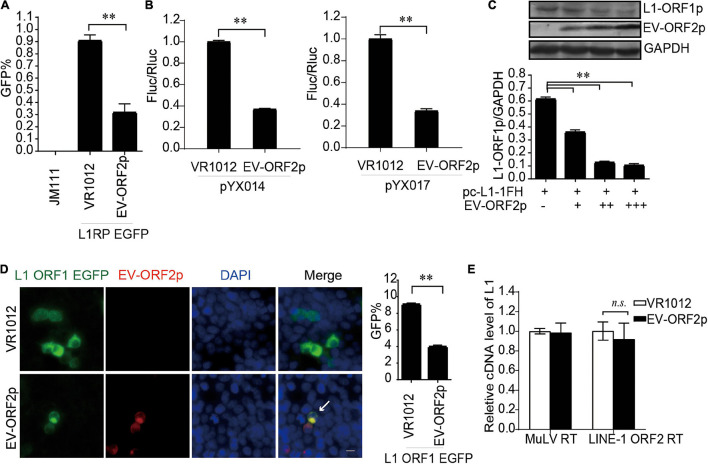
EV-ORF2p inhibited LINE-1 retrotransposition activity. **(A)** L1RP EGFP and VR1012/VR1012-EV-ORF2 plasmids were co-transfected into HEK 293T cells. Flow cytometry was performed after 4 days. The bar graph depicts the M ± SD of each experiment, performed in triplicate. The asterisks indicate statistically significant differences between groups by unpaired Student’s *t*-test (*p* < 0.01). **(B)** pYX014 or pYX017 constructs were transfected with VR1012 or VR1012-EV-ORF2p. The dual-luciferase assay was performed after 4 days. The VR1012 group was set to 1.0. The asterisks indicate statistically significant differences between groups by unpaired Student’s *t*-test (*p* < 0.01). **(C)** VR1012 or VR1012-EV-ORF2 plasmids (250, 300, and 350 ng) were co-transfected with pc-L1-1FH (250 ng). Cells were harvested after 48 h, and then immunoblotting was performed. **(D)** pEGFP-N1-ORF1-EGFP and VR1012/VR1012-EV-ORF2 plasmids were co-transfected. Immunostaining and fluorescence imaging were performed as described in the “Materials and Methods” 48 h post-transfection: scale bar, 20 μm. Bar graph represents the percentage of GFP-positive cells from flowcytometry. Data are represented as the mean ± SD, and the asterisks indicate statistically significant differences between groups by Unpaired Student’s *t*-test (*p* < 0.01). **(E)** LEAP assay and quantitative real-time PCR were performed as described in the “Materials and Methods” section. The bar graph of relative cDNA level of L1 represents reverse transcription efficiency of L1 ORF2p or MuLV. The relative cDNA level of MuLV RT was set to 1.0. The asterisks indicate statistically significant differences between groups by Unpaired Student’s *t*-test (*p* < 0.01).

Enterovirus protein ORF2p, also called UP, a newly identified viral protein encoded by certain enteroviruses, such as EV-A71 but not EV-D68, is essential for the release of viral particles in human gut cells (Guo et al., 2019; Lulla et al., 2019). Thus far, the intracellular functions of ORF2p are not fully understood. Here, we identified ORF2p as a virus-encoded suppressor of L1 transposition activity in different assays (Figures 6A,B). The presence of EV-A71 ORF2p expression was correlated with a significant decrease in L1 ORF1p expression (Figure 6C). This conclusion was supported by immuno fluorescence data (Figure 6D). However, EV-A71 ORF2p did not alter the reverse transcription activity of L1 ORF2p in L1 RNPs (Figure 6E). Hence, EV-A71 ORF2p impairs L1 ORF1p-dependent processes that are critical for L1 retrotransposition but not the L1 RNP RT activity.

## Discussion

**FIGURE 7:**
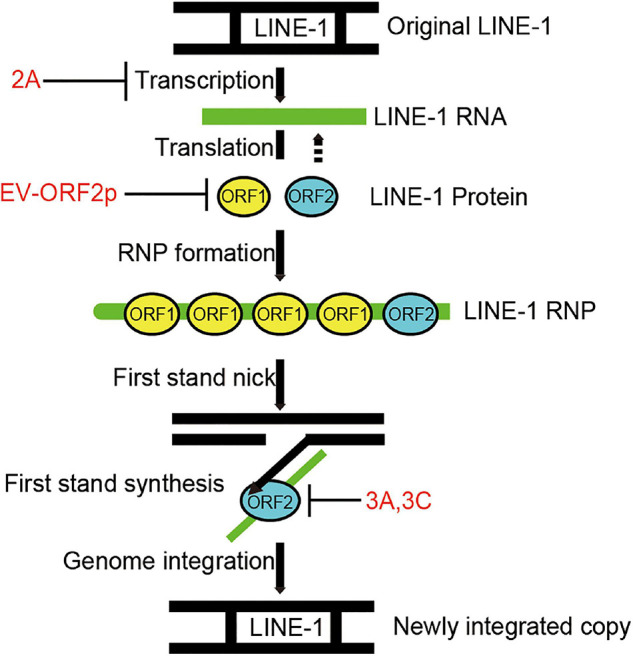
Diagram indicating that EV non-structural proteins 2A, 3A, 3C, and EV-ORF2p inhibit LINE-1 retrotransposition. LINE-1 is transcribed into LINE-1 RNA, and then the LINE-encoded proteins are translated. LINE-1 proteins interact with LINE-1 RNA to facilitate the assembly of LINE-1 ribonucleoproteins (RNPs). The LINE-1 ORF2p (endonuclease activity) nicks the bottom strand of the target site DNA. The LINE-1 ORF2p (reverse transcriptase activity) reverse transcribed the LINE-1 RNA using the 3’ hydroxyl group generated by LINE-1 ORF2p endonuclease activity as a primer. This process is a target-primed reverse transcription (TPRT). The DNA/RNA heteroduplex was converted to a DNA/DNA duplex and integrated into the target site. However, the underlying mechanisms remain unclear.

Long interspersed element 1s is an active human DNA parasite that shapes the human genome’s structure, function, and evolution (Brouha et al., 2003). Aberrant L1 transposition is deleterious to disturb genome stability and is associated with diverse diseases. Like viruses, L1 requires host co-factors to complete its life cycle. On the other hand, the host maintains a complex network system to suppress L1 transposition activity. Recent studies have intensively investigated host restriction factors for L1 inhibition to understand L1 surveillance and control in normal human cells (Finley, 2018). In this study, we have demonstrated that human enteroviruses belonging to the family Picornaviridae encode diverse viral inhibitors (2A, 3A, 3C, and ORF2p) that can broadly limit L1 mobility by acting on different L1 machinery components (Figure 7).

A series of reports have shown that many host antiviral factors, such as APOBEC3s, ZAP, SAMHD1, MX2/MxB, and TREX1, potently suppress the transposition activity of L1-and L1-dependent non-autonomous retrotransposons (Zhao et al., 2013; Moldovan and Moran, 2015; Goodier, 2016; Liang et al., 2016; Li et al., 2017). When invading host cells, viruses employ multiple strategies to counteract these intrinsic host defenses. Overcoming the functions of these antiviral factors by viruses should lead to the upregulation of L1 mobility. Unexpectedly, enterovirus infection strongly restricts L1 transposition activity, and enteroviruses encode four different proteins that interfere with the expression of L1 proteins and consequently the reverse transcription activity of L1 ORF2p. Studies have claimed that L1s are the endogenous drivers for activating innate immune responses (Zhao et al., 2018; Lee et al., 2019). In the present study, we used a luciferase-based IFN-β reporter system, and proved that EV-D68 can also suppress L1-triggered IFN-β production (Supplementary Figure 5). Our findings suggest that L1 transposition has an undesirable role in enterovirus replication.

Enterovirus 2A is a cysteine proteinase that is important for viral replication and pathogenesis. Besides viral proteinase 2A, which contributes to shearing viral poly-proteins, it can also cleave host proteins to facilitate viral infection (Ventoso and Carrasco, 1995; Hsu et al., 2007; Wang and Li, 2019). 2A-dependent cleaves the host translation initiation factor, eukaryotic translation initiation factor 4-gamma 1 (eIF4G1), which is correlated with the shutdown of global cap-dependent mRNA translation and stress granule formation in enterovirus-infected cells (Wu et al., 2014; Yang et al., 2018). It is known that the translation initiation activity of the 5’ UTR of L1 mRNA is critically dependent on the cap (Dmitriev et al., 2007). Overexpression of 2A dramatically suppressed the protein levels of L1 ORF1p (Figure 3C). Our results suggest that enterovirus 2A may inhibit L1 mobility through eIF4G1 cleavage-dependent translational blockage of L1 mRNA, which further emphasizes the modulatory roles of eIF4G1 in the transposition activity of endogenous retro-proteins.

Enterovirus 3A protein, a membrane-bound protein, can recruit acyl-coenzyme A-binding domain-containing 3 and phosphatidylinositol-4-kinase IIIβ to facilitate the viral assembly replication organelles (Lei et al., 2017a). These organelles provide docking sites for enterovirus polymerase 3D proteins to initiate RNA replication. In our study, viral protein 3A did not influence the expression and localization of L1 ORF1p but strictly suppressed the reverse transcription activity of L1 ORF2p. We thus suspected that viral protein 3A at viral RNA replication sites directly disrupts the functions of L1 ORF2p to prevent potential interference from intracellular RNA-binding proteins and maintain an optimal microenvironment for viral RNA synthesis. Future studies still need to characterize the detailed mechanisms of L1 inhibition by 3A.

Enterovirus 3C is another cysteine protease encoded by all the enteroviruses. Similar to 2A, 3C plays a determinant role in processing viral precursor polyproteins. Both 3A and 3C inhibit the RT activity in L1 RNP in a ORF1p-independent manner. As we could not obtain an effective antibody against L1 ORF2p, it is still necessary to investigate L1 ORF2 expression and the potential interaction between viral proteins and L1 ORF2p in future studies. Interestingly, we noted that the expression of 3A and 3C could increase the intracellular accumulation of L1 ORF1p proteins (Figures 4D, 5D and Supplementary Figure 7D), which may imply that virus-encoded proteins can also trigger the desuppression of L1 transposition during virus infection. Thus far, accumulating evidence suggest that a series of antiviral factors, such as APOBE3G, SAMHD1 (Kinomoto et al., 2007; Zhao et al., 2013), have been identified to be inhibitors of L1 activity. The counteraction of host antiviral factor by viral proteins would influence normal L1 mobility. Thus, virus engaged a complex network to modulate the L1 transposition. 3C also contributes to viral immune evasion through cleavage of the TAK1/TAB1/TAB2/TAB3 complex, TIR-domain-containing adapter-inducing interferon-β (TRIF), and IFN regulatory factor 7 (IRF7) to block cytokine production (Lei et al., 2010, 2013, 2014). 3C can cleave intracellular GSDMD to inhibit inflammasome-triggered interleukin-1 β release and pyroptosis (Lei et al., 2017b). Accumulating evidence has shown that LINE-1 drives IFN production and plays an important role in restricting LINE-1 propagation. However, innate immune inhibition by 3C did not increase the transposition activity of LINE-1 (Figure 5) but strongly inhibited L1 mobility by targeting the ORF2p function (Figure 5E). Further mechanistic studies are required to explain these phenomena. Our findings shed light on one viral protein strategy that has dual inhibitory functions both on L1 transposition and immune activation, in that extraordinary L1 activation-stimulated innate immunity is modulated by virus infection.

Recently, we and others identified a novel enterovirus protein, named ORF2p or UP, which is encoded by a second open reading frame in the genome of most enteroviruses (Guo et al., 2019; Lulla et al., 2019). Viral proteins contribute to viral release from human gut cells. Here, we uncovered another cell function of enterovirus ORF2p; the inhibition of retroelement LINE-1 transposition activity. Unlike 2A, 3A, and 3C, ORF2p only downregulated the protein levels of L1 ORF1p but did not affect L1 ORF2p function. In our previous studies, we found that EV-A71 ORF2p expression enhanced the activation of autophagy (Guo et al., 2019). The relationship between viral protein ORF2p-mediated autophagy and L1 ORF1p degradation is yet to be clarified.

Hence, we suggest that enteroviruses encoding diverse viral proteins can negatively modulate L1 mobility by targeting different processes of L1 transposition. The use of different viral proteins acting at different steps of L1 transposition together to control LINE-1 transposition reveals unignorable selection pressure from mobile DNA on virus replication.

## Data Availability Statement

Data from flow cytometry experiments was submitted to the FlowRepository. More information may be accessed via the following URL: flowrepository.org/id/RvFrXk8O1mykIRfUw1uiFiENBO6PdSQkCbXsCGOalFx8QcNOi7rFutb1zJJ3JNr7.

## Author Contributions

WW conceived and designed the experiments. YL, SS, and HG participated in multiple experiments. YL, SS, HG, ZZ, LZ, QY, YG, JN, and WW analyzed the data. YL, HG, and WW wrote the manuscript with help from all authors. All authors contributed to the article and approved the submitted version.

## Conflict of Interest

The authors declare that the research was conducted in the absence of any commercial or financial relationships that could be construed as a potential conflict of interest.

## Publisher’s Note

All claims expressed in this article are solely those of the authors and do not necessarily represent those of their affiliated organizations, or those of the publisher, the editors and the reviewers. Any product that may be evaluated in this article, or claim that may be made by its manufacturer, is not guaranteed or endorsed by the publisher.
