# Supplementary material for: Enterovirus Infection Restricts Long Interspersed Element 1 Retrotransposition
Source: Front Microbiol. 2021 Oct 18;12:706241. doi: 10.3389/fmicb.2021.706241 (PMC8559978; doi:10.3389/fmicb.2021.706241)
Supplement: Supplementary file 1 [file Data_Sheet_1.docx]

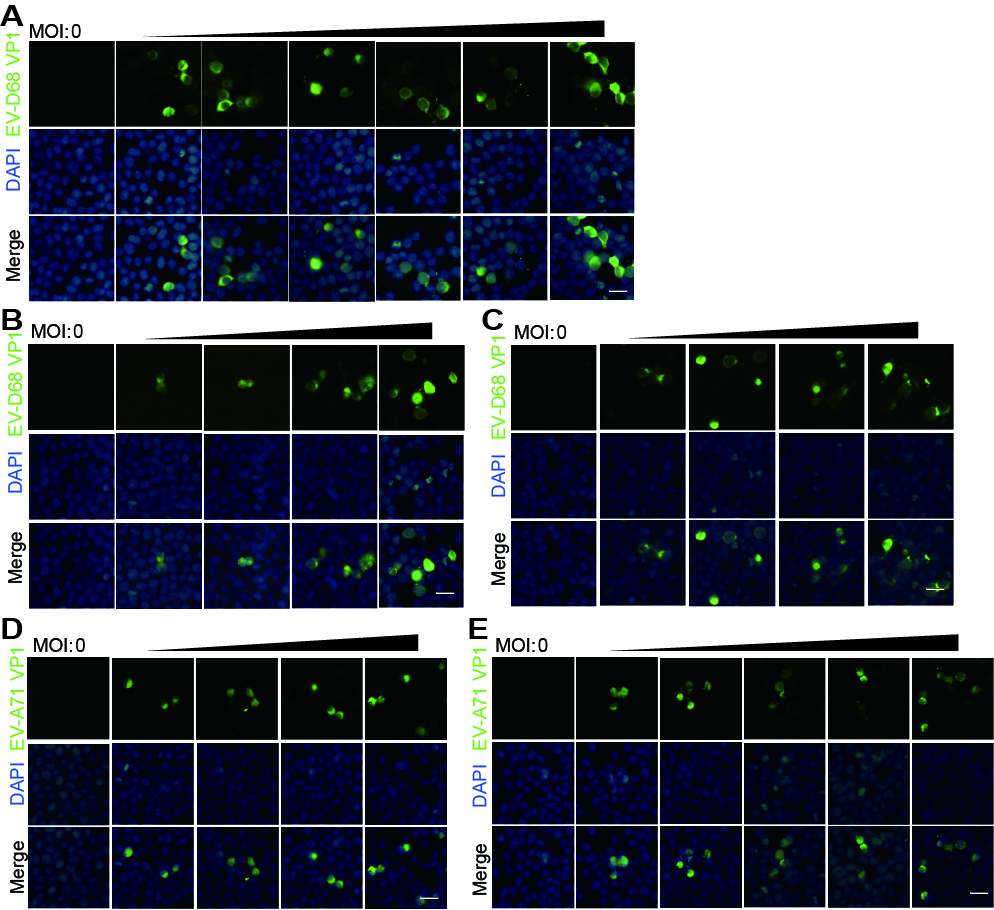


Fig. S1 Fluorescence Imaging of HEK293T cells infected with enterovirus

(A-C) HEK293 cells were infected with EV-D68 (Fermon) at increasing MOI of 0, 2×10^-5^, 5×10^-5^, 1×10^-4^, 2×10^-4^, 5×10^-4^, and 1×10^-3^ or EV-D68(US/MO/14-18947)/EV-D68(US/KY/14-18953) at increasing MOI of 0, 2×10^-5^, 5×10^-5^, 1×10^-4^, and 2×10^-4^. After 84h, the cells were fixed, permeabilized, and stained with EV-D68 VP1 antibody; nuclei were stained with DAPI. The cells were examined by fluorescence microscope. Scale bars equal 20 μm.

(D-E) HEK293T cells were infected with EV-A71(Anhui2007) at increasing MOI of 0, 2×10^-4^, 5×10^-4^, 1×10^-3^, and 2×10^-3^ or EV-A71(CC063) at increasing MOI of 0, 1×10^-3^, 2×10^-3^, 5×10^-3^, 1×10^-2^, and 2×10^-2^. After 84h, the cells were fixed, permeabilized, and stained with EV-D68 VP1 antibody; nuclei were stained with DAPI. The cells were examined by fluorescence microscope. Scale bars equal 20 μm.


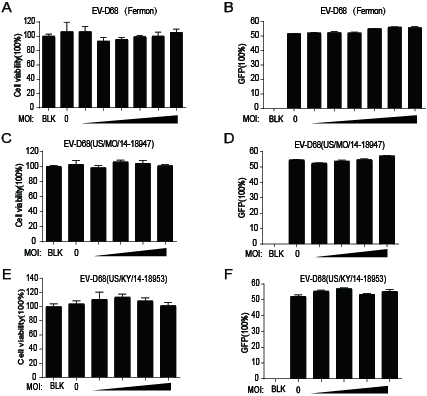


Figure S2. Cell viability and CMV promoter were not affected by enterovirus infection.

(A), (C), (E) Cells were infected with EV-D68 (Fermon) at increasing MOI of 0, 2×10^-5^, 5×10^-5^, 1×10^-4^, 2×10^-4^, 5×10^-4^, and 1×10^-3^ or EV-D68(US/MO/14-18947)/EV-D68(US/KY/14-18953) at increasing MOI of 0, 2×10^-5^, 5×10^-5^, 1×10^-4^, and 2×10^-4^. Thereafter, pre-infected HEK293T cells were seeded into 96-well plates 12 h post infection and cultured for 3 days. The cells were stained with MTS for 2 h. The absorbance was measured according to the protocol. The control-treated sample was set to 100%.

(B), (D), (F) pcDNA3.1-EGFP plasmids were transfected into HEK293T cells, and 12 h after transfection, cells were infected with EV-D68 (Fermon) at increasing MOI of 0, 2×10^-5^, 5×10^-5^, 1×10^-4^, 2×10^-4^, 5×10^-4^, and 1×10^-3^ or EV-D68(US/MO/14-18947) /EV-D68(US/KY/14-18953) at increasing MOI of 0, 2×10^-5^, 5×10^-5^, 1×10^-4^, and 2×10^-4^. EGFP-positive cells were determined by flow cytometry 4 days post transfection.


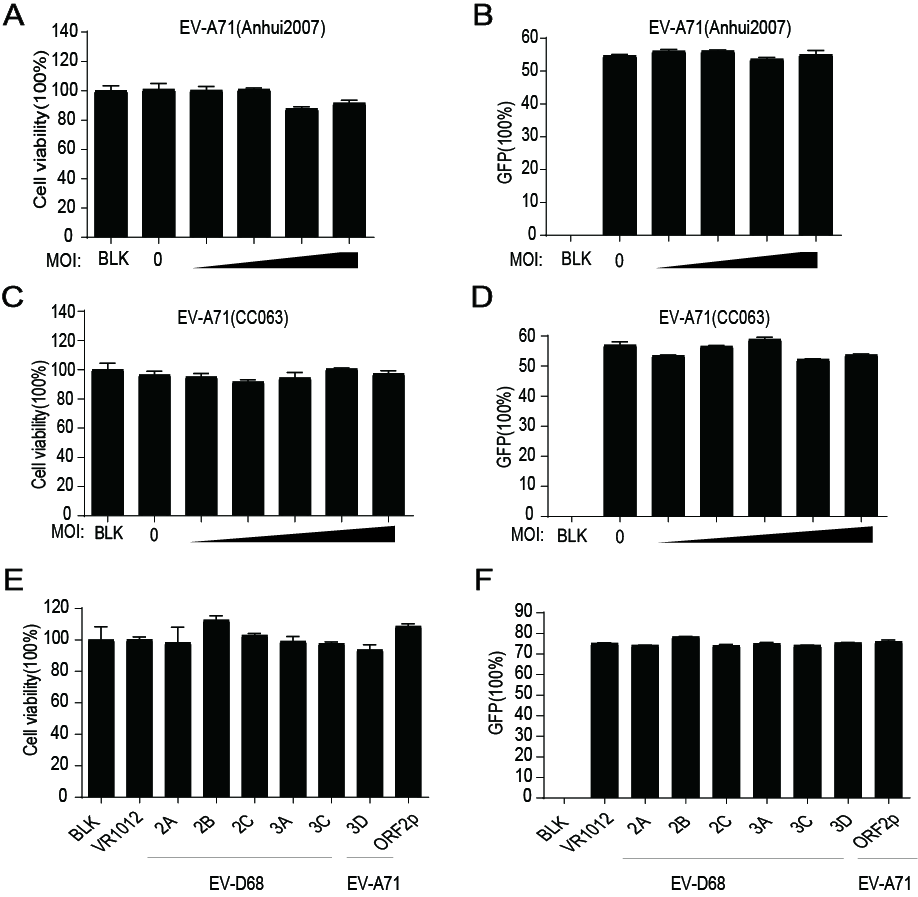


Figure S3. Cell viability and CMV promoter were not affected by enterovirus infection or viral proteins.

(A), (C) HEK293T cells were infected with EV-A71(Anhui2007) at increasing MOI of 0, 2×10^-4^, 5×10^-4^, 1×10^-3^, and 2×10^-3^ or EV-A71(CC063) at increasing MOI of 0, 1×10^-3^, 2×10^-3^, 5×10^-3^, 1×10^-2^, and 2×10^-2^. Thereafter, pre-infected HEK293T cells were seeded into 96-well plates 12 h after infection and cultured for 3 days. The cells were stained with MTS for 2 h. The absorbance was measured according to the protocol. The control-treated sample was set to 100%.

(B), (D) pcDNA3.1-EGFP plasmids were transfected into HEK293T cells, and 12 h after transfection, HEK293T cells were infected with EV-A71(Anhui2007) at increasing MOI of 0, 2×10^-4^, 5×10^-4^, 1×10^-3^, and 2×10^-3^ or EV-A71(CC063) at increasing MOI of 0, 1×10^-3^, 2×10^-3^, 5×10^-3^, 1×10^-2^, and 2×10^-2^. EGFP-positive cells were determined by flow cytometry 4 days post transfection.

(E) Cells pre-transfected with VR1012 or EV protein-expressing constructs were seeded in 96-well plates 24 h post-transfection and cultured for 3 days. The cells were then stained with MTS. The absorbance was measured according to the protocol. The control-treated sample was set to 100%.

(F) pcDNA3.1-EGFP plasmids and VR1012/EV protein-expressing constructs were co-transfected into HEK293T cells. EGFP-positive cells were determined by flow cytometry 4 days post transfection.


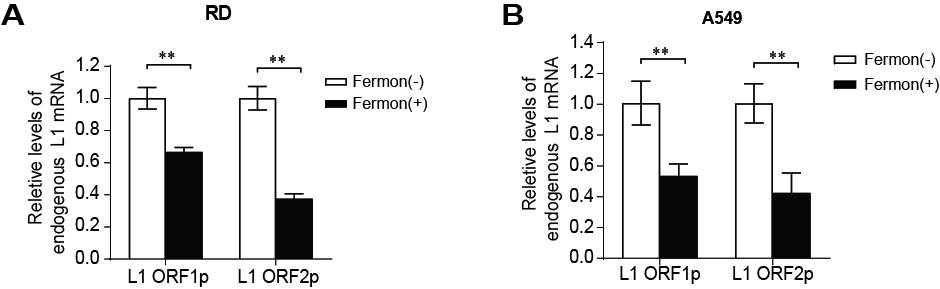


Figure S4. EV-D68 infection supresses LINE-1 expression in RD and A549 cells.

RD cells(A) and A549 cells(B) were seeded and infected with EV-D68 (Fermon) at an MOI of 0.011. Cells were harvested 48 h post infection and the total RNA was isolated. The qRT-PCR was performed using L1 ORF1p, L1 ORF2p, and GAPDH specific primers. The asterisks indicate statistically significant differences between groups by Unpaired Student's t-test (p< 0.01).


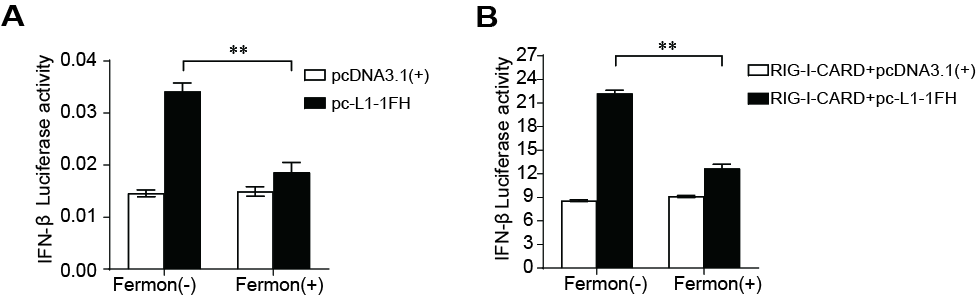


Figure S5. EV-D68 suppresses L1-triggered IFN-β production.

(A) The IFNβ-luc plasmids driven by the IFN-β promoter expressing Firefly luciferase were transfected into HEK 293T cells. Renilla luciferase plasmids transfected into each well were used for normalisation. One hour post transfection, the cells were infected with EV-D68 (Fermon) at an MOI of 0.011. Dual-luciferase assays was performed 48 h post transfection. The bar graph shows the ratio of Firefly and Renilla luciferase.

(B) The IFNβ-luc plasmids were co-transfected with RIG-I-CARD expressing plasmids to trigger basic Firefly luciferase activity. Renilla luciferase plasmids transfected into each well were used for normalisation. One hour post transfection, the cells were infected with EV-D68 (Fermon) at an MOI of 0.011. Dual-luciferase assays was performed 48 h after transfection. The bar graph shows the ratio of Firefly and Renilla luciferase. The asterisks indicate statistically significant differences between groups by Unpaired Student's t-test (p< 0.01).


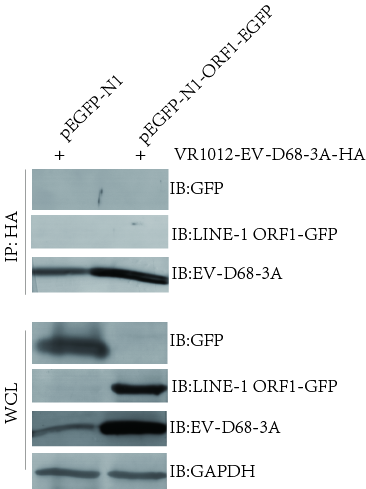


Figure S6. EV-D68 3A does not interact with L1 ORF1p

pEGFP-N1-ORF1-EGFP and EV-A71-3C plasmids were co-transfected. For HA-tagged 3A and EGFP-tagged L1 ORF1p immunoprecipitation, the protein-expressing cells were harvested and were lysed at 4°C for 1 h. Anti-HA affinity matrix was mixed with prepared cell lysates and incubated at 4°C overnight. The eluted proteins were subsequently analyzed by Western blotting.


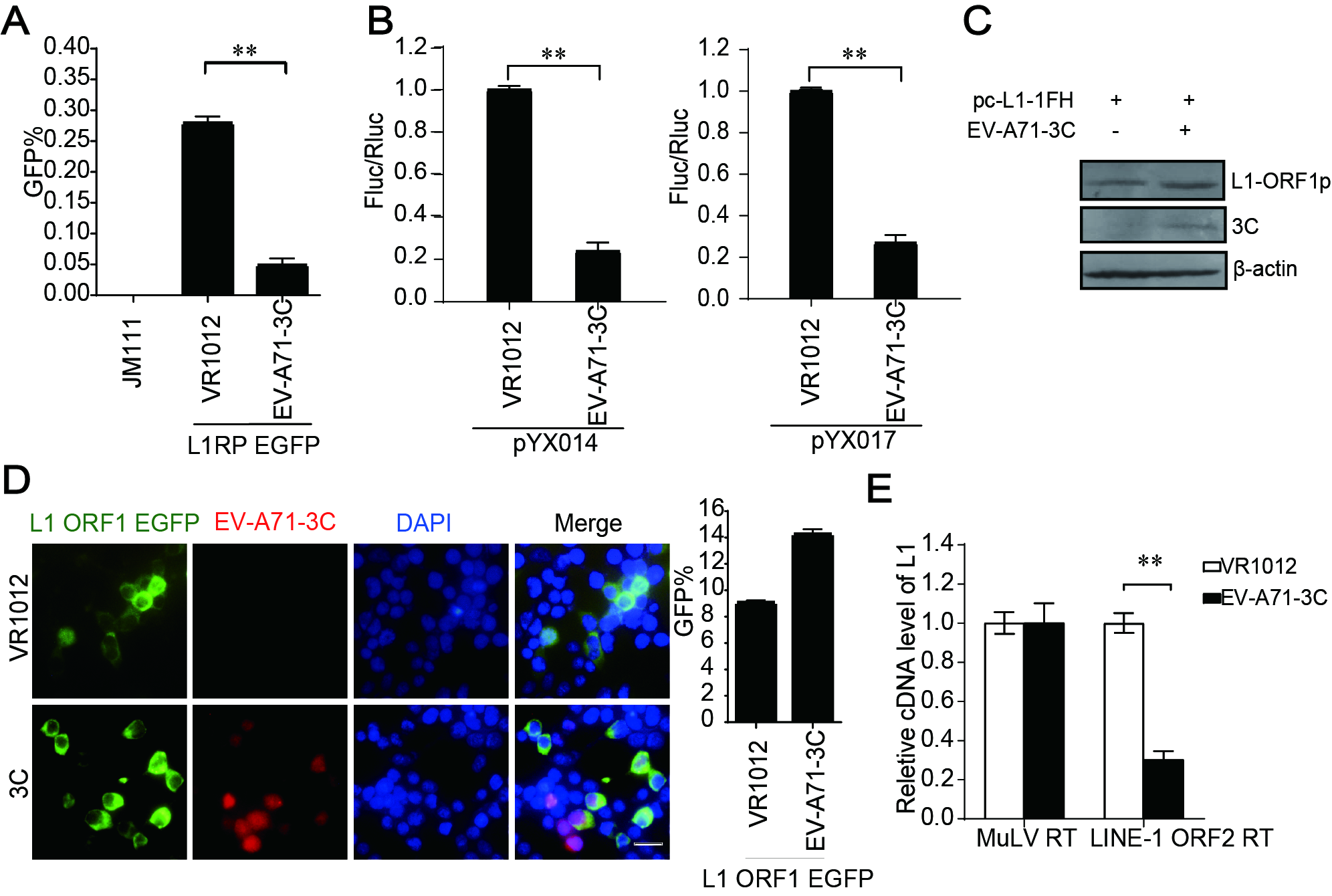


Figure S7. Suppression of LINE-1 retrotransposition by EV-A71 3C

(A) L1RP EGFP plasmids and VR1012/ EV-A71-3C plasmids were transfected into HEK 293T cells. JM111 was used as a negative control. Flow cytometry was performed 96h post-transfection. The bar graph depicts the M ± SD of each experiment, performed in triplicate The asterisks indicate statistically significant differences between groups by Unpaired Student's t-test (p< 0.01).

(B) HEK 293T cells were transfected with pYX014 or pYX017 constructs together with VR1012 or EV-A71-3C. Then dual-luciferase assay was performed 4 days post-transfection. The VR1012 transfected sample was set to 1.0. The asterisks indicate statistically significant differences between groups by Unpaired Student's t-test (p< 0.01).

(C) VR1012 or EV-A71-3C plasmids were co-transfected with pc-L1-1FH. Western blot was performed after 48h.

(D) pEGFP-N1-ORF1-EGFP and EV-A71-3C plasmids were co-transfected. Immunostaining and fluorescence imaging were performed as described in the Methods 48 h post-transfection: scale bar, 20 μm. Bar graph represents the percentage of GFP-positive cells from flowcytometry. The asterisks indicate statistically significant differences between groups by Unpaired Student's t-test (p< 0.01).

(E) LEAP assay and quantitative real-time PCR were performed as described in the Methods. The bar graph of relative cDNA level of L1 represents reverse transcription efficiency of L1 ORF2p or MuLV. The relative cDNA level of MuLV RT was set to 1.0. The asterisks indicate statistically significant differences between groups by Unpaired Student's t-test (p< 0.01).
